# Geographic Object-Based Analysis of Airborne Multispectral Images for Health Assessment of *Capsicum annuum* L. Crops

**DOI:** 10.3390/s19214817

**Published:** 2019-11-05

**Authors:** Jesús A. Sosa-Herrera, Moisés R. Vallejo-Pérez, Nohemí Álvarez-Jarquín, Néstor M. Cid-García, Daniela J. López-Araujo

**Affiliations:** 1 Laboratorio Nacional de Geointeligencia, CONACYT-Centro de Investigación en Ciencias de Información Geoespacial, Aguascalientes 20313, Mexiconcid@centrogeo.edu.mx (N.M.C.-G.); djlopez@centrogeo.edu.mx (D.J.L.-A.); 2 Coordinación para la Innovación y Aplicación de la Ciencia y la Tecnología (CIACYT), CONACYT-Universidad Autónoma de San Luis Potosí, San Luis Potosí 78000, Mexico; vallejo.pmr@gmail.com

**Keywords:** precision agriculture, *Capsicum annuum*, GEOBIA, remote sensing

## Abstract

Vegetation health assessment by using airborne multispectral images throughout crop production cycles, among other precision agriculture technologies, is an important tool for modern agriculture practices. However, to really take advantage of crop fields imagery, specialized analysis techniques are needed. In this paper we present a geographic object-based image analysis (GEOBIA) approach to examine a set of very high resolution (VHR) multispectral images obtained by the use of small unmanned aerial vehicles (UAVs), to evaluate plant health states and to generate cropland maps for *Capsicum annuum* L. The scheme described here integrates machine learning methods with semi-automated training and validation, which allowed us to develop an algorithmic sequence for the evaluation of plant health conditions at individual sowing point clusters over an entire parcel. The features selected at the classification stages are based on phenotypic traits of plants with different health levels. Determination of areas without data dependencies for the algorithms employed allowed us to execute some of the calculations as parallel processes. Comparison with the standard normalized difference vegetation index (NDVI) and biological analyses were also performed. The classification obtained showed a precision level of about 95% in discerning between vegetation and non-vegetation objects, and clustering efficiency ranging from 79% to 89% for the evaluation of different vegetation health categories, which makes our approach suitable for being incorporated at *C. annuum* crop’s production systems, as well as to other similar crops. This methodology can be reproduced and adjusted as an on-the-go solution to get a georeferenced plant health estimation.

## 1. Introduction

Modern precision agriculture (PA) technologies can help to optimize the use of crops’ input resources, such as fertilizers, pesticides, and water, just to name a few, reducing in general an unsuitable use of them, and consequently increasing the crop’s production [1,2]. The employment of remote sensing (RS) and geographic information systems (GIS) tools have proved to be very effective for agricultural activities [2,3]. In recent years, aerial observations for crop management using UAVs have increased considerably, specifically for yield prediction [4,5], disease detection [6], weed identification [7], crop quality [8], and so on. Compared with satellite remote sensing and aerial images captured by manned aircraft, UAVs are among the most cost-effective technologies, providing high flexibility of use, and low cost for the data acquisition [9]. Some of the disadvantages of using UAVs for RS tasks are that they cannot cover large areas, UAV sensor models are limited, and more image preprocessing work is required compared with spectral data obtained by satellites, which are equipped with more sophisticated sensors, and deliver products of very large area imagery, where calibration and many corrections have already been done [2].

Given the increased availability of very high resolution (VHR) imagery and enhanced computing power, geographic object-based image analysis (GEOBIA) has become a significant tool to interpret clusters of pixels into meaningful information in the form of geometric objects. There exist a myriad of applications where the information given by GEOBIA becomes relevant, as can be found in [10].

Despite the above-mentioned technology progress, many of the cited methodologies have not been extended as standard production procedures yet. Among the factors that could influence such limitation, we can cite that some of them only consider individual pixels, without taking into account the surrounding elements, while others involve calculations for which too much computation effort is needed when they are extended to large crop areas, mainly because of the very high resolution image processing and artificial intelligence (AI) operations involved, even requiring, in many cases, the use of specialized big data techniques to perform a large amount of computations in specific time windows to provide the answers sought for certain agricultural applications [11].

Even when VHR image acquisition has become simpler and widely spread, research on evaluating crops health via autonomous image processing remains scatter. Some methodologies related to the one described in this paper can be found in [12,13,14]. In one of the earliest works [12], multispectral images taken by airborne sensors and spectral data were used to evaluate the stage of infection of two tomato fields resulting in the positive identification of infected tomatoes at pixel image level. In [13], aerial multispectral imagery was used to evaluate hail damage in potato crops based on defoliation. Health assessment of lettuce plants is performed in [14] from multispectral data obtained with a camera positioned in close range as input for an expectation maximization cluster analysis with a Gaussian mixture model. However, that procedure cannot be used to evaluate a whole crop. Vegetation health evaluation by analyzing airborne data to detect diseases at infected plants implementing AI methods have also been done in previous works. Some of them are based on clustering [15,16,17], back propagation networks [18], neural networks [19], color histograms [20], and support vector machines [21,22], just to name a few. However, the main difference of the present research and the cited works, consists in the modeling of plant structures by employing GEOBIA techniques to obtain segments associated to the shapes of branches and leaves, then establishing plant boundaries at contiguous vegetation pixels. As a result, groups of segments representing plants originated at individual seeding points are obtained. These groups are employed to define a reliable discrete plant health index by averaging spectral modes of segments considered as belonging to the same seeding point.

The main contributions of the proposed scheme are that by the use of UAVs, portable multispectral devices, and an algorithmic pipeline we are able to distinguish up to five vegetation health levels at individual seeding points in a crop. The plant health indexing proposed here, is designed to deal with variations of spectral signatures presented by different leaves of the same plant, mixed reflectance features at leave edges, irregular plant shapes, incorrectly classified segments, and errors at the estimation of plant locations. This is mostly done by averaging features present at groups of bordering segments, instead of adjacent pixels. We achieved this in a practical manner by combining scalable algorithms for high speed segmentation, classification, and clustering, while also introducing a new health indexing approach that involves the geometric distribution and shape of vegetation objects. Training and validation for supervised classification is assisted by the use of a custom portable spectrometer that automatically geolocates spectral signatures at points where the samples are taken. Besides the training and validation steps, the rest of the workflow is performed automatically. Some of the calculations involved can be executed as parallel processes by defining regions with no data dependencies, allowing by this to obtain results shortly after all the needed data were gathered. Our procedure is demonstrated with a study case with which we validated the efficiency of the developed methodology. The experiment was carried out in a production crop of *C. annuum* in the Mexican central highlands region.

## 2. Materials and Methods

The plant health estimation method described here, makes use of portable multispectral technology to obtain VHR images in order to properly identify objects of interest at crop fields using several AI algorithms. Additionally, individual spectral signatures of objects of interest and soil samples were taken to be analyzed in laboratory. The procedure presented can be applied to a wide variety of crops in order to evaluate plant health levels in a parcel, by means of the analysis of high resolution multispectral images which could be acquired by UAVs, manned flights, or even by satellites. To describe them in a very specific manner, we choose a study case consisting on the plant health evaluation of a parcel of *C. annuum* crop. The corresponding details are described in the following subsections.

### 2.1. Study Area

The area of study consisted of half a hectare of *C. annuum* crop located at $22^{\circ }50^{\prime }11^{\prime \prime }$ N, $102^{\circ }40^{\prime }18^{\prime \prime }$ W 2205 m, in the community of Morelos, Zacatecas State, Mexico. For the purpose of this research, local production practices were followed, as we sought to develop an on-the-go technique for vegetation health assessment that can easily be escalated to larger field extensions. Detailed descriptions of the agricultural practices employed are described in [23]. Plant cultivation started in February, 2018 with groups of 3 seeds raised in seedling starter trays with a sterilized coconut coir substrate. These plants were transplanted in soil after six weeks of growth, with a distance of 0.3 m between seeding points disposed in double rows with 0.5 m and 1.5 m of internal and external separation, respectively. Drip irrigation was supplied 2 days a week. A reference crop parcel with wide phenotypic variations among plants was selected to capture different stages of plant decaying. Evaluation of the cultivars began in July 2018.

### 2.2. Data Acquisition and Image Preprocessing

**Figure 1:**
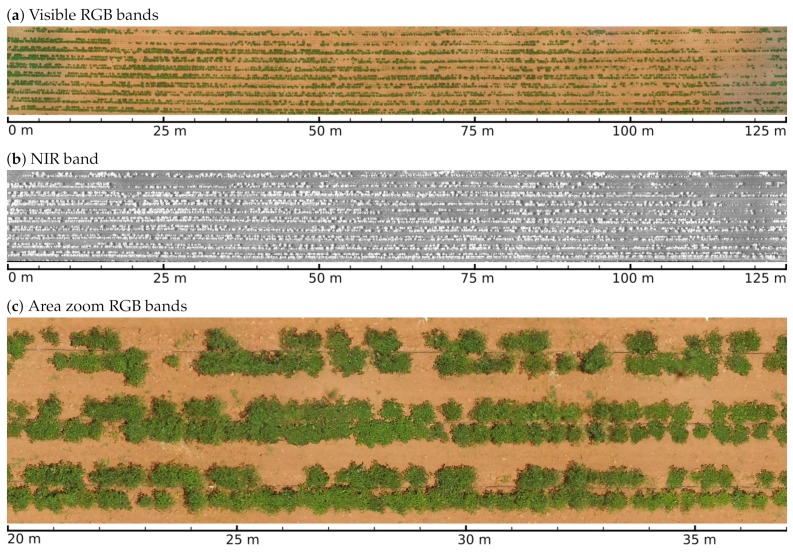
Orthomosaics of the study parcel.

For the data acquisition, two Phantom III Standard^®^ (SZ DJI Technology Co., Ltd., Shenzhen, China) multirotor UAVs, adapted with a Parrot Sequoia^®^ (Parrot SA, Paris, France) multispectral camera were used to obtain multispectral images with the resolution needed to identify crop objects. Automated flight missions were programmed using the Pix4D Capture^®^ (Pix4D, Lucerne, Switzerland) software. ground control points (GCPs) were placed every 3 m along ploughing direction. The flights were performed at 15 m above ground level covering an area of 5000 m${}^{2}$ at a speed of 10 m/s. In this way, 15 GB of multispectral imagery were gathered with an overall resolution of 2 cm/px in the wavelengths green (G) 550 nm, red (R) 660 nm, red edge (RE) 735 nm, and near infrared (NIR) 790 nm, with respective bandwidths of 40, 40, 10, and 40 nm. Image pixel levels had a 14-bit precision in GEOTIFF format. RGB images with a standard camera were also taken. Generation of RGB and multispectral point clouds and orthomosaics were executed with the Pix4D Mapper^®^ (Pix4D, Lucerne, Switzerland)) software. After this preprocessing, resolution dropped to 4 cm/px and the effective area covered by orthomosaics was reduced to 2250 m${}^{2}$, in order to discard border images and non-crop objects. The Parrot Sequoia^®^ multispectral camera integrates an irradiance sensor that was calibrated with a Micasense^®^ (MicaSense Inc., Seattle, WA, USA) reflectance panel before each flight mission of the UAVs. The firmware of the multispectral camera writes irradiance calibration measurement parameters at the EXIF headers in the GEOTIFF files, along with other photogrammetric data, inside each image corresponding to G, R, RE, and NIR bands. Polynomial coefficients for vignetting correction, camera pose angles, and GPS coordinates are also registered in the EXIF headers. The Pix4D Mapper^®^ software works by looking at this information to automatically convert digital numbers (DN) into radiance values, and to generate the respective orthomosaics. Poncet et al. show in [24] that this setup is able to produce radiometric indices with an accuracy comparable to some empirical calibration methods. Note that radiometric correction and camera pose parameters are not written for images from the RGB sensor. Therefore, only G, R, RE, and NIR orthomosaics are used in the calculations of the workflow described below. Figure 1 shows non calibrated RGB and calibrated NIR orthomosaics of the study parcel, it also shows a portion of a zoomed area, to give a visual representation for the detail level of the orthomosaics. A digital elevation model (DEM) was also generated from the same point clouds used to create the orthomosaics.

Spectral signatures for objects of interest were also taken in the field. To this end, we designed a low cost portable spectrometer, which was used to obtain georeferenced signatures of different crop samples. It also features an interface with a mobile application that makes possible to visualize a real-time graphic representation of reflectance curves and calibration parameters. We implemented the portable spectrometer with a printed circuit board based on the C12880MA (Hamamatsu Photonics K.K., Shizuoka, Japan) sensor which detects 288 wavelengths. C12880MA signals were collected trough a digital general purpose input output (GPIO) port using a generic ATMega328^®^ (Atmel Corp., San Jose, CA, USA) microcontroller which sends signal level values to a mobile device via an universal serial bus (USB) connection. A custom software written in Java programming language for controlling the C12880MA sensor with a mobile device was developed using the Android Studio SDK^®^ (Google Inc., Mountain View, CA, USA) environment. The software developed was responsible for dynamic sensor calibration, user interface controls, real-time graphic representation of reflectance spectra, geolocation, and data storage. The C12880MA chip has a typical full width at half maximum (FWHM) of 12 nm and a maximal FWHM of 15 nm [25]. The spectral resolution of the C12880MA sensor is not linear throughout its operational bandwidth, but rather can be modeled by the polynomial

(1) $$p\left(x\right)=A_{0}+B_{1}x+B_{2}x^{2}+B_{3}x^{3}+B_{4}x^{4}+B_{5}x^{5}$$

where *x* is the index of the pixel measured as an output signal level, and $A_{0}$, $B_{1}\ldots B_{1}$ are coefficients determined at factory tests [26]. The specific coefficients for the device used are shown in Table 1. The Java program developed for the mobile device interface, uses these coefficients to interpret the signals generated by the C12880MA chip, and rounds the results of the evaluation of p(x) to the nearest integer to graphically represent the measured reflectance values at the mobile device screen.

**Figure 2:**
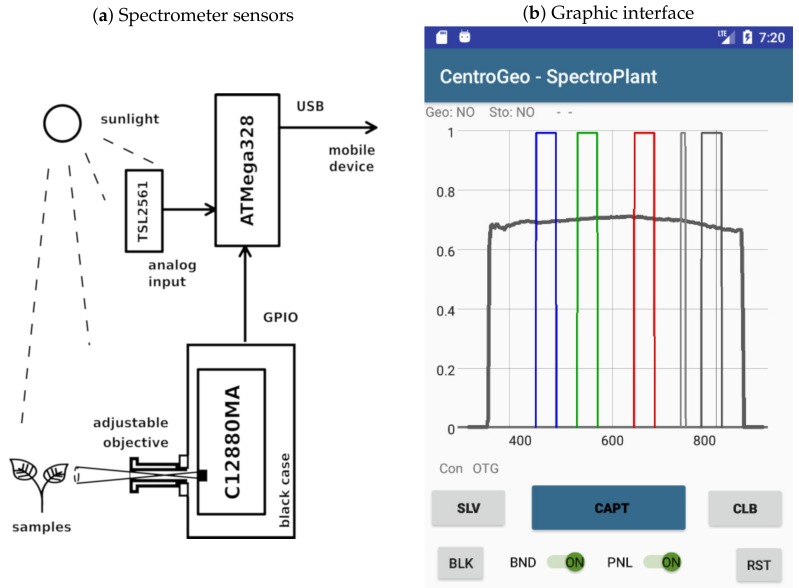
Portable spectrometer with mobile device interface.

An auxiliary TSL2561 (AMS AG, Premstaetten, Austria) sensor was added as an analog input to the ATMega328^®^ microcontroller to perform dynamic calibration of the C12880MA chip output levels under different sunlight conditions. The calibration reference taken was the Micasense^®^ reflectance panel for wavelengths between 360 nm and 850 nm in bandwidth center increments of 1 nm. Figure 2a shows a diagram of the device implemented to obtain georeferenced spectral signatures on field, Figure 2b presents a view of the program interface. The gray plot shown in this figure corresponds to the coefficients from the reflectance panel for dynamic calibration, the vertical bars represent the B, G, R, RE, and NIR bands detected by the multispectral camera. The portable spectrometer was built in order to be able to obtain in field a set of georeferenced spectral signatures of portions of crop objects with enough spatial resolution to appear as endmember pixels in the VHR multispectral images gathered by the UAVs. The spectral signatures were taken at regularly spaced points, and then mapped to image segments belonging to vegetation to assign the labels to segments used for training and validation of a supervised classifier.

### 2.3. Algorithmic Pipeline

The processing stack proposed in this paper with the aim of identifying plant health states, starts with an a priori definition of health categories defined by *C. annuum* phenotype features evaluated after six months of plant growth. Five health categories were determined, based on their principal phenotypical characteristics: Height (cm), canopy surface $\left(m^{2}\right)$, and on the percentage of observed change in leaf morphology; namely curly leaves, spotted leaves, and yellowing (chlorotic leaves). The respective plant health category labels are identified as HL1,…,HL5 from the lowest to highest as shown in Table 2. Figures presented at this table were determined by visual inspection an counting of features at seeding points inside crop areas designated for training and validation of the algorithms, and some manual labor was required to obtain such data. In this way, we have a classification of plants based on a combination of different observed features related to the presence of plant disease symptoms, or otherwise, their absence.

**Figure 3:**
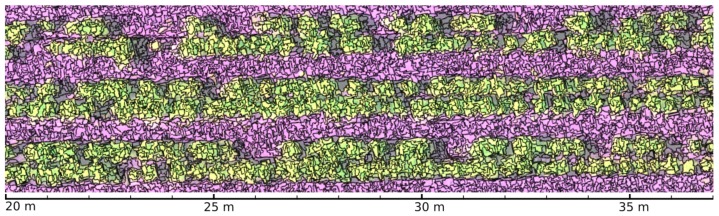
Large scale mean shift segmentation (LSMSS) segmentation of a multispectral crop’s image composed by the green (G), red (R), red edge (RE), and near infrared (NIR) bands.

The next step consists in applying the large scale mean shift segmentation (LSMSS) algorithm [27] on the multispectral images. It is convenient to have the input images rectified and stitched as an orthomosaic as described in the preprocessing section. LSMSS was presented by Michel et al. as an efficient version of the spatial extension of the mean shift segmentation (MSS), a non-parametric clustering procedure by Comaniciu and Meer [28]. The MSS algorithm takes an image with pixels $X=\{x_{i}:\;i=1,\ldots ,n\}$ and produces another image where the pixel values of $Z=\{z_{i}:\;i=1,\ldots ,n\}$ have been assigned to be equal to the local maxima of the clusters found by the iterative procedure on $j=1,\ldots ,j_{max}$ defined by:

(2) $$\begin{array}{cc} y_{i,1} & =x_{i}\\ y_{i,j+1} & =\frac{\sum_{x_{k}\in N\left(y_{i,j}\right)}K\left(x_{k}-y_{i,j}\right)x_{k}}{\sum_{x_{k}\in N\left(y_{i,j}\right)}K\left(x_{k}-y_{i,j}\right)}\\ z_{i} & =(x_{i,j}^{s},y_{i,j}^{r}) \end{array}$$

where $y_{i,j}$ is the *j*-th approximation to the mode corresponding to pixel $x_{i}$, N(x) represents the neighboring pixels of x at spatial range $h_{s}$ and spectral range $h_{r}$, *t* stands for a convergence threshold and K(x) is a kernel function. In [28] a radially symmetric kernel *K* derived from the Epanechnikov kernel [29] is used, although Gaussian kernels are also applicable. Superscripts *s* and *r* refer to spatial and spectral components, respectively. After the last iteration, adjacent $z_{i}$ points converging to the same modal values are labeled as part of the same segment. The use of LSMSS here is introduced to help us to model the boundaries between contiguous vegetation pixels, as the shapes of the segments generated in vegetation areas tend to follow the contours of leaves and branches of plants. The implementation of the stable version of [27] provided by the Orfeo Toolbox (OTB) library was used to apply LSMSS in our procedure. The spatial and spectral parameters were fixed to $h_{s}=5$ and $h_{r}=15$, respectively, based on plant object sizes and their spectral variations. Figure 3 shows a section of the crop’s multispectral images segmented by the LSMSS algorithm in a background of false colors combining G, R, RE, and NIR spectral bands.

After executing the LSMSS segmentation, the next step performs a supervised classification operation. We selected the maximum likelihood classifier (MLC), a commonly used procedure in many remote sensing applications [30,31]. In MLC, the probability for an element s with a feature vector ω to belong to a class C is given by:

(3) $$P\left(C|\omega \right)=\frac{P(\omega |C)P(C)}{\sum_{C^{\prime }}P\left(\omega |C^{\prime }\right)P\left(C^{\prime }\right)}$$

where P(ω∣C) is the class conditional density for ω, P(C) is the a priori probability of any element to belong to class C, and the divisor is the likelihood of observing ω as data. Assuming a multivariate Gaussian distribution, the class conditional density P(ω∣C) can be modeled by the following logarithmic likelihood function:

(4) $$ln\left(P\left(\omega ,C\right)\right)=-\frac{1}{2}{\left(\omega -\mu \right)}^{T}\Sigma^{-1}\left(\omega -\mu \right)-\frac{1}{2}ln\left(\left|\Sigma \right|\right)-\frac{N}{2}ln\left(2\pi \right)$$

where *N* is the number of classes, μ is the mean of the distribution, and Σ is the covariance matrix. MLC chooses the values for μ, and the entries of Σ that maximize ln(P(ω,C)) by equating its derivatives to zero. An element s is assigned to class C if:

(5) $$P\left(C|\omega \right)>P\left(C^{\prime }|\omega \right)\;\;\forall \;C^{\prime }\neq C$$

For the input of MLC, segments obtained at the LSMSS stage were taken as elements s and the spectral modes $y_{i,j}^{r}$ in Equation (2) were used as feature vectors ω. Then MLC was applied to segments instead of pixels. Each of the training segments with class labels belonging to vegetation and soil in selected crop areas were identified and geolocated. In the case of plants, the corresponding segments were cataloged into one of the HL1,…,HL5 health levels. There were some areas at which soil and vegetation signatures where heavily mixed, mainly at the edges of plants where soil was partially covered by vegetation and their silhouettes. Segments in these areas were labeled as shadows. Additionally, the spectral signatures of objects of interest were registered with the spectrometer device described in a previous section, which also provided latitude and longitude coordinates through the GPS unit integrated in the mobile device, allowing in this way to obtain georeferenced labels of segments for identified objects. The MLC operation was executed by making use of the System for Automated Geoscientific Analyses (SAGA) [32]. In particular, MLC was chosen as it gave the best precision among other supervised classifiers from the SAGA library, including minimum distance to means classifier (MDM) [33], spectral angle mapping (SAM) [34], nearest neighbor classifier (NNC) [35], and the parallelepiped classifier (PC) [36]. A comparison of the average precision of these methods at the classification of all labels for the segments obtained by LSMSS is shown at the results section in Table 3.

**Figure 4:**
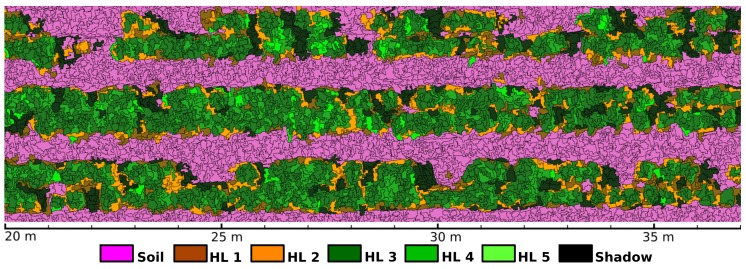
Maximum likelihood classification of segments.

Figure 4 shows an area at which the MLC step has been applied. In this figure, segments classified with the label ‘Shadow’, correspond to adjacent pixels with very low reflectance levels that could not be matched with vegetation or soil.

Once each segment had been labeled, all polygons representing vegetation were grouped together and saved into a file with shapefile (.shp) format for its processing in QGIS [37]. In order to calculate seeding point center locations, we considered the pixels under these regions and estimated the number and position of inscribed circles of diameter d=0.3 m, which is the mean distance of seeding points separation, by doing so we took an approach of a clustering problem [38]. A convenient option for solving this grouping step is the employment of the KMeans algorithm [39]. With the purpose of preserving the separation distance specified by *d*, we set the number of classes *k* in the KMeans algorithm to $k=4a/\pi d^{2}$, where *a* denotes the area of the polygons enclosing each connected vegetation region. To efficiently determine which pixels have to be considered while evaluating *k* and *a*, instead of querying polygon boundaries of segments directly from the shapefiles, we applied the function ‘cv2.connectedComponents’ included in the OpenCV library [40] to assign connected component labeling (CCL) markers [41] on a binary mask consisting of pixels corresponding to vegetation areas. This binary mask can be built by joining the pixels inside vegetation segments labeled at the classification step. Then, by calculating KMeans centroids, the grouped areas were not limited to round shapes, but they were rather defined by a two-dimensional Voronoi tessellation [42]. KMeans is not used for classification purposes in this step of the pipeline. It is instead applied to approximate the center locations of regions formed by segments belonging to vegetation emerging from the same point. The election of the parameter *k* is thus defined for the recognition of segments belonging to same plants. The implementation of the KMeans clustering algorithm was the included in OpenCV. A custom script was written in Python programming language [43] to feed the OpenCV API functions with the geographic coordinates of each vegetation region. It is worth noting that in the KMeans clustering, only spatial information of the pixels inside vegetation segment regions was used, and spectral data were discarded. That is, only the $x_{i,j}^{s}$ vectors of Equation (2) were involved in this phase.

**Figure 5:**
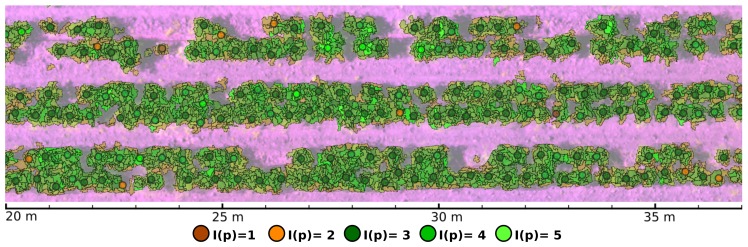
Health indices I(p) for estimated seeding points.

With the estimation of seeding point center locations, the next stage of the pipeline consists in determining health indices associated to each seeding point. We propose an index based on the assignation of HL1,…,HL5 classes defined in Table 2, mapped to the corresponding integer value in the set {1,…,5}. Then, at each detected seeding point *p*, a neighborhood B(p,r) with center in *p* and radius *r*, is considered to define a set of segments $S_{p,r}$ as:

(6) $$S_{p,r}=\left\{s\in A:s\cap B\left(p,r\right)\neq \emptyset \right\}$$

where A is the set containing all segments generated by LSMSS. The health level index for a seeding point is then defined by:

(7) $$I\left(p\right)=\frac{1}{n}\sum_{s\in S_{p,r}}I\left(s\right)$$

with I(s) denoting the health level associated to the classification made by MLC for segment s, and *n* representing the cardinality of $S_{p,r}$. By defining this indexing scheme, we aim to compensate for centroid estimation errors, spectral signature mixing of soil and vegetation at leaf and branch edges, irregular plant shapes, and reflectance variations of plant leaves originated at the same seeding point. Figure 5 shows health levels I(p) for estimated seeding points obtained by applying Equation (7) with r=7.5 cm over a crop’s image region. Note that many vegetation segments classified as soil that can be appreciated at Figure 5 were composed of decayed foliage that was no longer performing photosynthetic processes. In consequence, they presented very low reflectance values at NIR wavelengths. Some other segments were definitely misclassified. The number of erroneously labeled segments are shown in the form of a confusion matrix at Table 4 in the results section.

**Figure 6:**
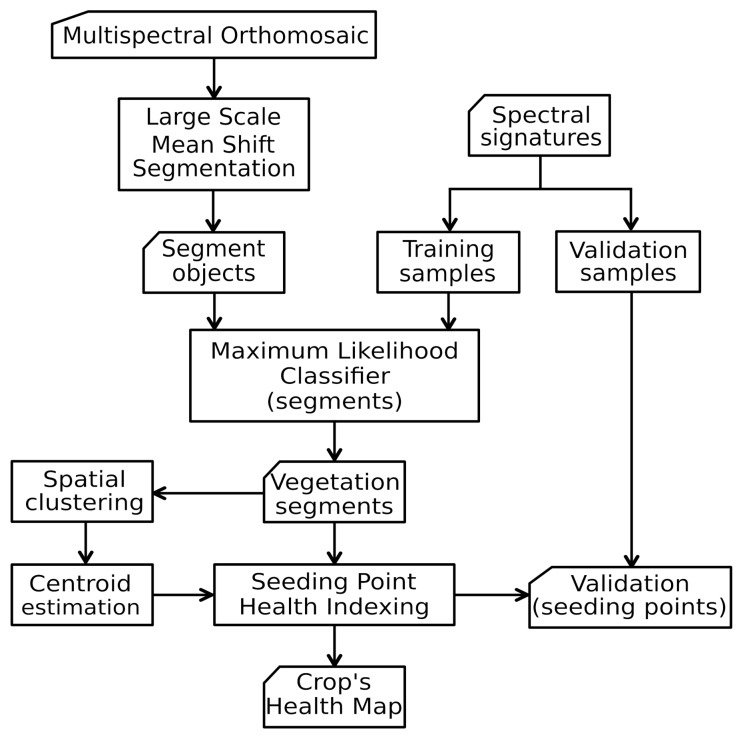
Proposed pipeline for obtaining seeding point health level maps from high resolution multispectral images.

Figure 6 depicts a flowchart for the entire pipeline starting with preprocessed multispectral images into the form of orthomosaics. Besides the stitching and orthorectification of the images, the rest of the processing pipeline was performed using open source software and libraries, complemented with custom scripts. A concise step-by-step description of the procedure is presented in Algorithm 1.

**Algorithm 1:**Geobia-based steps for plant health indexing of crops
**Input:** Multispectral orthomosaic. Georreferenced spectral signatures.

**Output:** Plant health indexing and location of seeding points.
**Step 1**:Segmentation. Execute the LSMSS algorithm on the multispectral orthomosaic of the region of interest, using spatial and spectral range parameters according to the size of the objects to be identified and their spectral variations.**Step 2**:Training. Create a table with the characteristics that properly define the most representative categories. Take the average spectral signatures of objects at such categories. Geolocate these signatures and assign labels to the corresponding segments on specific training areas.**Step 3**:Segment classification. Apply the supervised machine learning algorithm MLC to classify the segments obtained by LSMSS. Other supervised algorithms can also be used at this step, as long as they provide a good accuracy level.**Step 4**:Clustering. Calculate plant or seeding point locations by the use of a clustering algorithm over pixels belonging to vegetation segments. For this, consider the average plant size and separation of seeding points. The KMeans algorithm applied on the spatial components of pixels can do the work required by this step.**Step 5**:Indexing. Evaluate the health index at each seeding point by assigning a numeric value to every category determined at Step 2. Then, take the average of the corresponding values of the pixels inside all segments that intersect a neighborhood of an specific radius from each seeding point by using Equation 7.**Step 6**:Validation. Estimate the precision of the health indices calculated at Step 5 by using georeferenced spectral measurements and observations on specific validation areas. This will give an insight on the precision of the results obtained.


### 2.4. Training and Validation Area Distribution

**Figure 7:**
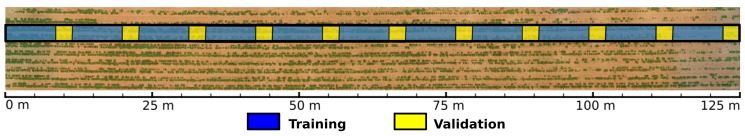
Training and validation areas.

A portion of 420 m${}^{2}$ of the crop area was visually inspected, and spectral firms of soil and vegetation objects were taken and geolocated with the device above described. This extension covered 1808 seeding points and was divided into adjacent training and validating regions. These regions were starting from the third double row in order to avoid edge effects, and were distributed along two rows allowing samples to be taken from different longitudinal regions of the crop. Figure 7 shows the placement of the training and validation polygons. All the plants inside training and validation areas fell into one of the health levels HL1,…,HL5 defined in Table 2.

In this way, 15,000 segments obtained by the LSMSS algorithm were labeled with the aid of the QGIS selection tool and a custom Python script to record them as components of objects corresponding to soil, shadows, and vegetation with health levels from HL1 to HL5 as defined in Table 2. Such segments acted as training inputs for the MLC algorithm. Another 5000 segments were used to evaluate the accuracy of MLC. Besides, registers of health levels from 440 seeding points at the validation regions served for the verification of the final health level indices.

### 2.5. Performance and Parallel Execution

To get a measure for the performance of the proposed workflow, the computational complexity of the algorithms involved can be done by tracing the main operations inside their loops [44]. Step 1 is based on MSS, which relies on kernel density estimation [28]. According to Equation (2) this can be performed in $O_{S}=O\left(mn_{p}\right)$ time, with $n_{p}$ being the number of pixels in the image, and *m* the size of the neighborhood taken to evaluate kernel modes. Step 2 is a manual process assisted by the portable spectrometer and GIS tools. When the classification at Step 3 is performed with MLC, the temporal complexity is determined by Equation (4), from which we can see that the sum of the computational complexities associated to each term of the right side of Equation (4) multiplied by the number of classes $n_{c}$ is:

(8) $$n_{c}\left(O\left(d_{s}^{2}n_{t}\right)+O\left(1\right)+O\left(d_{s}^{2}n_{t}\right)+O\left(n_{t}\right)\right)=O\left(n_{c}d_{s}^{2}n_{t}\right)$$

where $n_{t}$ is the number of training segments and $d_{s}$ is the dimension of the covariance matrix Σ at Equation (4) which is given by the number of bands used in the classification step. Additionally, probability comparisons at Equation (5) are done in $O(n_{c}n_{s})$ time, where $n_{s}$ is the number of segments produced in Step 1. Therefore, the complexity $O_{C}$ for the classification stage have the form:

(9) $$O_{C}=O\left(d_{s}^{2}n_{t}\right)+O\left(n_{c}n_{s}\right)$$

Step 4 involves the use of the clustering algorithm KMeans, for which finding an optimal solution is known to be a NP-hard problem [45]. However, the iterative Lloyd’s procedure [46] gives approximate solutions of the KMeans problem for $n_{v}$*d*-dimensional vectors and *k* clusters in complexity time $O(idkn_{v})$, with *i* representing the number of its iterations. In the present work, only spatial components of the vegetation associated pixels $n_{v}$ were considered at the clustering stage, thus, the corresponding dimension is d=2. Moreover, the number of iterations was limited to i=20 to manage the clustering complexity in the worst case. The time complexity of the CCL algorithm used for determining the number of clusters *k* is $O(n_{p}^{2})$[41], therefore, the complexity $O_{K}$ for the clustering stage is given by:

(10) $$O_{K}=O\left(idkn_{v}\right)+O\left(n_{p}^{2}\right)≅O\left(kn_{v}\right)+O\left(n_{p}^{2}\right)$$

Step 5 queries $n_{l}$ location vectors of the detected seeding points against $n_{sv}$ segments containing vegetation pixels. Spatial queries in this work were scripted in Python for the POSTGIS database running under QGIS. A spatial query on *N* elements has a typical time complexity of O(logN)[47], consequently the complexity $O_{I}$ of the queries needed to evaluate I(p) at the indexing step can be expressed as:

(11) $$O_{I}=O\left(n_{l}n_{sv}logn_{sv}\right)$$

Hence, time complexity $O_{T}$ of the entire workflow can be expressed as:

(12) $$\begin{array}{cc} O_{T} & =O_{S}+O_{C}+O_{K}+O_{I}\\ \; & =O\left(mn_{p}\right)+O\left(d_{s}^{2}n_{t}\right)+O\left(n_{c}n_{s}\right)+O\left(kn_{v}\right)+O\left(n_{p}^{2}\right)+O\left(n_{l}n_{sv}logn_{sv}\right)\\ \; & ≅O\left(n_{p}\right)+O\left(n_{t}\right)+O\left(n_{s}\right)+O\left(kn_{v}\right)+O\left(n_{p}^{2}\right)+O\left(n_{l}n_{sv}logn_{sv}\right). \end{array}$$

Taking into account that *m*, $d_{s}$, and $n_{c}$ are small and fixed, and considering the relations:

(13) $$n_{p}>n_{v}\gg n_{s}>n_{sv}>n_{l}\;\mathrm{and}\;n_{s}\gg n_{t}$$

we have that the complexity in Equation (12) is dominated by the term $O(n_{p}^{2})$, associated to spatial clustering operations. Therefore:

(14) $$O_{T}≅O_{K}≅O\left(n_{p}^{2}\right).$$

**Figure 8:**
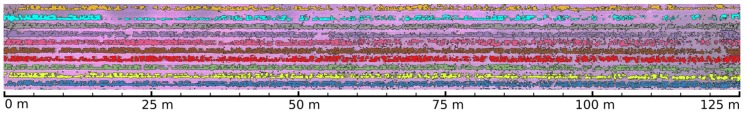
Area division for parallel execution. Each color represents vegetation areas evaluated by the same computer process.

To speed up the execution of the spatial clustering, the vegetation regions were divided into polygons belonging to the same plowing row, by querying their spatial coordinates. It is worth to note that by partitioning vegetation areas in this way, there is no data dependency for the input vectors to the KMeans algorithm, as all of them belong to different unconnected pixel areas. Therefore, it was possible to send the corresponding areas of each row to a different parallel execution process, as presented in Figure 8, which was implemented by making use of the ‘multiprocessing.Pool’ interface included in the standard Python libraries. The scheduling scripts were run on a HP Z440 workstation with an Intel Xeon E5-2630 CPU (Intel Corp.) at 2.4 GHz, with 32 GB of RAM, featuring 8 physical cores and 16 logical cores. In order to obtain performance metrics of the parallelization of Step 4, five executions of the workflow were performed over the same orthomosaic varying the number of parallel processes $N_{p}$ and averaging execution times obtained using the *time* package included in Phyton. Amdahl’s law [48] was used to determine the speedup $S(N_{p})$, the portion of code *P* that was effectively executed in parallel, given a constant workload, and the complementary non-parallelizable portion 1−P with the expression:

(15) $$S\left(N_{p}\right)=\frac{1}{\left(1-P\right)+P/N_{p}}$$

### 2.6. Phytosanitary Soil Analysis

With the purpose of searching for correlations between canopy reflectance properties and plant root health states, microbiological analysis of soil samples were performed at uniformly sparsed points in the training and validation areas, in order to search for pathogens that might be affecting the crop. Previous studies reported that in regions near the area where the experiment was conducted, one of the main pathogen that affects *C. annuum* crops is *Phytophthora capsici* Leonian [49]. Then, soil samples were analysed under laboratory conditions to determine the presence of fungal pathogens. The analysis included soil samples of 100 g that were collected from alternating rows and two *C. annuum* plants were considered per point, the process was repeated each 20 m until complete 14 samples were gathered throughout a field area of 120 m long with four double-rows of 3 m width, a space of 10 m to the borders of the crop was left between the initial and final soil sample. The soil samples were taken near plant roots at 10 cm below ground surface and were individually stored in sterile plastic bags. Then, they were labeled and transported for processing. Soil isolation and identification of microorganism strains procedures were executed as follows. These soil samples were homogenized and 1 g (3 replications/sample) was deposited in a Falcon^®^ tube. Next, 10 mL of deionized water was added and were put in a vortex mixer for 10 minutes (Maxi Mix II, Thermo Scientific). The resulting suspension was used to prepare a 10:1 dilution and 100μL were seeded in Petri dishes with V8-Agar medium supplemented with PCNB (100μg/mL), benomyl (25μg/mL), hymexazole (25μg/mL) and ampicillin (500μg/mL) [50]. The Petri dishes were incubated at $28{\;}^{\circ }$C under dark conditions and were inspected every 12 hours under a stereomicroscope (10X) to detect the mycelial growing of colonies. Observations were counted to register the colony forming units per gram (CFU/g) of soil. The mycelial growths were transferred to V8-Agar medium for maintenance and observation. The identification was done considering its morphological characteristics [51], and pure isolated strains were molecularly analyzed by extraction of genomic DNA using the CTAB method [52]. PCR amplification was done analyzing the internal transcriber spacer (ITS) regions of fungal ribosomal DNA (rDNA) with oligonucleotides ITS1 (5${}^{\prime }$-tccgtaggtgaacctgcgg-3${}^{\prime }$) and ITS4 (5${}^{\prime }$-tcctccgcttattattgatatgc-3${}^{\prime }$) according to White et al. [53], under the following PCR conditions: $94{\;}^{\circ }$C 5 min, 30 cycles $94{\;}^{\circ }$C 1 min, $55{\;}^{\circ }$C 45s, $72{\;}^{\circ }$C 2 min, and $72{\;}^{\circ }$C 2 min final extension. PCR products of expected sizes (650–700 bp) were purified and sequenced. The BLASTn algorithm was used to search the NCBI GenBank database [54] to confirm taxonomical assignment.

## 3. Results

### 3.1. Object-Based Image Analysis

**Figure 9:**
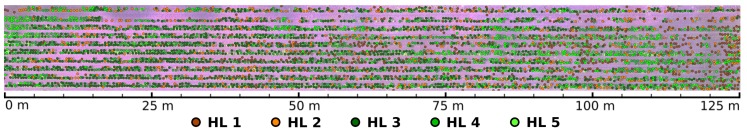
Automated seeding point detection and classification on the study area.

**Figure 10:**
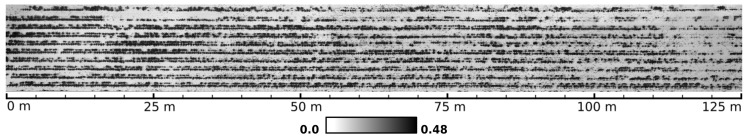
Normalized difference vegetation index (NDVI) levels for the study area.

The procedure described in the previous section allowed us to obtain an automated detection and classification of seeding points in a parcel of a *C. annuum* crop. Figure 9 shows an image of the resulting identification based on a discrete vegetation health index on high resolution multispectral images obtained by small quadrotor type UAVs. NIR and R spectral bands were used to obtain an orthomosaic of the area representing the NDVI index, evaluated as:

(16) $$NDVI=\frac{NIR-R}{NIR+R}$$

which is shown in Figure 10.

**Figure 11:**
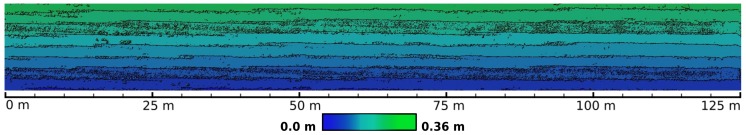
Digital elevation model map of the study parcel.

Moreover, the images taken included several camera poses for overlapping regions which permitted to construct a DEM from the respective point clouds. This resulted in a graphic representation of terrain elevation as shown in Figure 11.

**Figure 12:**
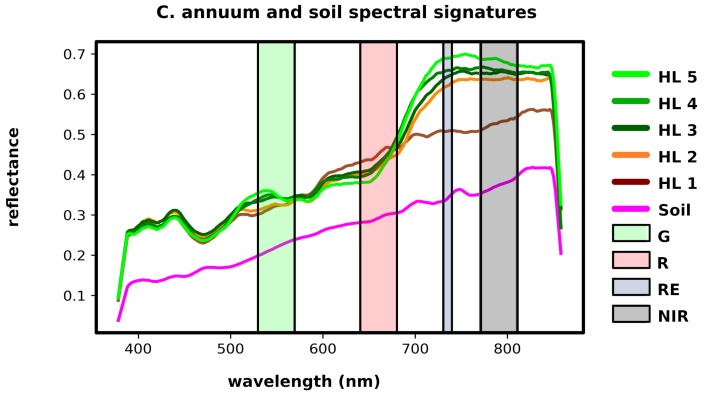
Class average spectral signatures of soil and plants classified at different health levels. Color strips represent band ranges captured by the multispectral camera.

The portable spectrometer device allowed us to record signatures of plants with geographic coordinates attached. This data were used to train the MLC and to validate its results as well as the results given by the proposed health indexing method. Such training data were complemented with records obtained by visual inspection on field for the specific areas described in Section 2.4. Figure 12 depicts the graphs of the average spectral signatures corresponding to 20 plant samples associated with each defined class of vegetation and background soil. Vertical color strips in Figure 12 represent the spectral bands captured by the multispectral camera.

Average precision for the classifications of LSMSS generated segments using several supervised methods is presented in Table 3. Additionally, the classification of segments given by MLC was validated on the areas specified in Section 2.4. Table 4 presents the confusion matrix obtained for such evaluation. The classes considered correspond to soil, shadows, and five vegetation levels HL1,…,HL5. In this table, columns stand for the predicted classes and rows are the ground truth data registered for validation regions. In the same way, Table 5 shows the confusion matrix for the classification of health indices I(p) of seeding points verified at the validation areas. The precision:

(17) precision=(TP)/(TP+TN)

where TP and TN stand, respectively, for the number of true positives and true negatives, is also shown in Table 5 for each health index level in the last column. The symbol ∅ represents the seeding points at which no plant have survived after four months of being transplanted. The total seeding point counts with their corresponding health indices I(p) obtained by the automated process divided by plowing rows labeled as $R_{1},\ldots ,R_{10}$ from top to bottom over the studied crop parcel is displayed at Table 6.

Running times of individual automated steps from the proposed workflow, using a single processor are presented in Table 7. The performance metrics for the parallelization of the clustering operations, varying the number of parallel processes $N_{p}$, and the parameters involved in Equation (15) are shown in Table 8. Summarizing data from these tables, we can see that total workflow execution took 8.89 h to complete using a single processor, and 1.5 h using 10 processes, each of them running the clustering step on different parts of the orthomosaic. Different areas assigned to each parallel process are drawn in Figure 8.

### 3.2. Phytosanitary Soil Analysis

**Figure 13:**
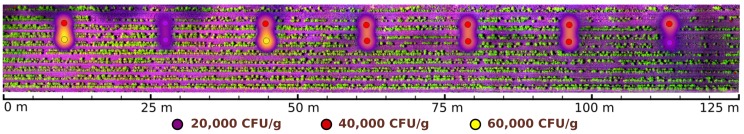
Colony forming unit (CFU)/g of *Mortierella* sp. detected at sampled sites.

The main microorganism isolated with the selective medium used was the zygomycet fungus *Mortierella* sp. For this, white fungal colonies were isolated from soil, they showed rosaceous growth patterns with cenocitic and torulous mycelium. The isolates produced sporangiosphores of 7–10 μm in size, straight and unbranched, the zygospores were of 23–78 μm in size. The molecular analysis confirmed the morphometric results in agreement to the DNA sequences, these showed 98 to 99% of similarity and *e*-value of 0.01, conforming to *GenBank* data. The CFU counts indicated that *Mortierella* has a uniform distribution in field and inoculum concentrations varying from 20,000 to 60,000 CFU/g soil. Figure 13 displays a map of the CFU/g concentration at sampled sites. From the samples taken, we were not found traces of other pathogens that could be associated to the disease symptoms presented by the plants. Other fungus and bacteria microorganisms commonly associated to plant diseases, including *P. capsici*, were not present in enough quantities to form significant CFU count levels.

## 4. Discussion

The combination of the GEOBIA and machine learning techniques in the form of the proposed pipeline for the analysis of VHR multispectral images, by the integration of LSMSS, MLC, spatial clustering, and geometry consensus; allowed us to get an estimate of plant health levels of single seeding points over all the crop’s surveyed area. A map of the outcomes of such levels is exemplified in Figure 9. For comparison matters, we calculated the widely used NDVI index map, illustrated in Figure 10. It can be noted that the GEOBIA approach gave us more specific details about plant health conditions, as well as location and distribution of such features, than those that can be extracted from the NDVI map. The spectrometer device developed for these research allowed us to have geolocated spectral signatures that were fed to scripts to label training and validating area segment objects. The class average signatures of such objects are presented in Figure 12, where the most notable reflectance variations between plants classified at different health levels occurred on the RE, and NIR band ranges. Background soil spectral signature is very different from that belonging to vegetation, in consequence, NDVI clearly distinguishes between vegetation and background soil. Nevertheless, as we are sampling mostly the same species of vegetation, plants assigned to distinct health level classes present similar reflectance values at individual bands. Unlike NDVI, which only uses R and NIR bands, the proposed pipeline makes use of the four bands available on the multispectral images, which enabled the algorithms to perform a further health level hierarchy division inside the vegetation segment class. Such division produced five categories based on phenotypic plant features. It is important to note that some of the symptoms, as leaf curliness, chlorosis, and leaf spots, are not clearly distinguishable in the orthomosaics, even at their highest resolution of 4 cm/px as can be seen in Figure 1c. However, the average spectral signatures are affected in a way that makes possible for the MLC algorithm to discern mean spectral values for segments and uniquely assign them to their respective category. The segment class definitions is also supported by the class average spectral signatures shown in Figure 12.

The exactitude of segment classification by the MLC stage shown in Table 4 in the form of a confusion matrix, can be regarded as being very good when it comes to distinguish from soil and vegetation segments. Soil segments were taken as shadows in a small percentage. In a few rare cases soil segments were confused with vegetation with very low health values, this is, with segments containing mainly wilted or dried leafs. For the vegetation segments cases, we can see that the confusion of segments at different health levels also presented a low portion of errors for the classification. These errors appeared mainly among segments with close health levels, and no segment with high health index I(p) was mistaken for soil or shadow. The confusion matrix and precision figures shown in Table 5 correspond to the validation of I(p) levels for discrete seeding points detected by the clustering process. We see again that precision drops occur only when comparing points with similar I(p) values, and incorrect assessments between healthy and unhealthy plants are almost null. Then, we consider this automated process to be reliable for determining discrete vegetation health indices when it comes to discern between decaying and vigorous plant growth.

The way in which the spectral VHR images are obtained by using the UAVs, allowed us to produced a set of point clouds, that is used not only for creating the corresponding orthomosaics, but also to generate a DEM map, as depicted on Figure 11, where a certain height difference from top to bottom rows can be seen. Besides, ground level remains almost constant along each row. Slightly elevated regions defining lines formed by plant placement sites are depicted as black contour lines. Such regions form a barrier that obstructs irrigated water flow from higher to lower rows. Thus, terrain elevation is not causing local water accumulation areas. Height difference between contiguous rows never exceeded 4 cm. On the other hand, median plant height was about 45 cm, thus, most reflectance variance came from plant leaf shadows rather than from terrain slope. This lead us to introduce the class labeled as ‘Shadows’, to group segments with relatively lower reflectance values. From the biological analysis, we see at Figure 13 that the peak CFU concentration values of *Mortierella* sp. are located at zones surrounded by healthy plants according to the evaluated I(p) indices. As reported by studies presented in [55,56], *Mortierella* sp. presence is associated to conditions that promote crop growth and limit the proliferation of certain native soil pathogens. However, collected soil samples were very few, so strong conclusions cannot be derived from this. Even so, the proposed algorithmic pipeline allowed us to successfully obtain georeferenced indices detailing plant health conditions and their distribution. One possible limitation for the method described here to work in more general cases, is that separation between plants or seeding points must be known beforehand, and such distance has to be relatively uniform with respect to individual plant dimensions. Such conditions however, are very common to find in many modern production crops.

## 5. Conclusions

In this article we presented a new technique to evaluate the health state of *C. annuum* plants located at individual seeding points. It is based on geographic object image analysis using high resolution multispectral images obtained by UAVs. Our scheme employed phenotypic traits to define plant health categories that are fed to a supervised machine learning classifier.

Generated plant health maps were validated by spectral signatures taken in field, and by laboratory analyses of microbiological samples which could be used to get a better understanding of the possible causes of the observed disease symptoms. The efficiency of the calculated indexes was quite reliable as confusion matrices showed that most matching errors occurred among adjacent health condition levels.

The entire stack of algorithms described here can be applied to a wide variety of crops, exhibiting uniform phenotypic traits and a homogeneous spatial distribution of plants. The resulting data can be used to aid farmers in decision making throughout the production cycle of the crop, as the spatial analysis and the extraction of distribution patterns of plant health by means of the presented method can uncover growth anomalies for their early corrective management. The workflow followed is intended to replace visual in situ plant health assessment and manual data registration, which are time consuming, expensive and error prone. Another interesting feature of our approach is that it allows the use of multispectral imagery for the detection of features validated with spectral signature measurements in such a manner that it can be easily replicated with commercial devices with a similar precision.

On the other hand, there are some limitations for the proposed method. We can cite for example that it can only be applied to monoculture crops with regular distribution of plants, for which average size, separation distance, and spectral signature variations are previously known. It might be also difficult to extend the procedure to very large fields due to computing restrictions and data gathering issues inherent to small UAVs technology.

## Figures and Tables

**Table 1:** Factory determined spectral resolution coefficients for the C12880MA sensor SN17I00621.

$A_{0}$	$B_{1}$	$B_{2}$	$B_{3}$	$B_{4}$	$B_{5}$
$3.17926058\times 10^{2}$	2.69726310	$-1.32864319\times 10^{-3}$	$-5.39983110\times 10^{-6}$	$-7.95625547\times 10^{-10}$	$2.01449675\times 10^{-11}$

**Table 2:** Plant health categories based on phenotypic trait combinations.

	HL1	HL2	HL3	HL4	HL5
**Height** (cm)	<30	30 to <40	40 to <50	50 to 60	>60
**Surface** $\left(m^{2}\right)$	<0.10	0.10 to <0.15	0.15 to <0.20	0.20 to 0.25	>0.25
**Curly** %	>60	30 to <60	15 to <30	10 to <15	<10
**Spotted** %	>20	10 to <20	<10	0	0
**Chlorotic** %	>40	20 to <40	<20	0	0

**Table 3:** Comparison of average precision among different supervised classifiers for LSMSS segment labeling.

Classifier	MDM	PC	SAM	NN	MLC
**Precision**	0.6104	0.6358	0.6625	0.7816	0.9255

**Table 4:** Confusion matrix for the maximum likelihood classifier (MLC) segment classes.

Truth	Soil	Shadow	HL1	HL2	HL3	HL4	HL5
**Soil**	2209	73	4	2	0	0	0
**Shadow**	48	235	12	0	0	0	0
**HL1**	17	10	781	6	0	0	0
**HL2**	1	4	3	401	17	0	0
**HL3**	0	0	0	21	533	14	3
**HL4**	0	0	0	8	11	409	9
**HL5**	0	0	0	0	2	7	158

**Table 5:** Confusion matrix (left columns) and precision (right column) for evaluated health indices of seeding points.

Truth	∅	1	2	3	4	5	Precision
∅	189	6	3	0	0	0	0.9545
**1**	2	39	0	0	0	0	0.9512
**2**	1	0	25	2	0	0	0.8929
**3**	0	0	8	79	13	0	0.7900
**4**	0	0	0	7	47	3	0.8246
**5**	0	0	0	2	5	26	0.7879

**Table 6:** Total seeding point health index I(p) counts for the study parcel divided by plowing rows $R_{1},\ldots ,R_{10}$ from top to bottom.

I(p)	$R_{1}$	$R_{2}$	$R_{3}$	$R_{4}$	$R_{5}$	$R_{6}$	$R_{7}$	$R_{8}$	$R_{9}$	$R_{10}$	Sum
∅	556	593	469	487	439	455	400	429	443	399	4570
1	55	50	45	61	109	46	97	89	62	51	665
2	80	50	44	52	42	37	56	56	68	69	554
3	120	99	173	135	161	179	165	179	194	238	1643
4	57	67	109	102	97	98	109	106	76	92	913
5	32	41	60	63	52	85	73	41	53	51	551

**Table 7:** Execution time of automated steps using a single processor.

	Segmentation	Classification	Clustering	Indexing
**Time (min.)**	18.72	3.09	508.76	2.6

**Table 8:** Performance metrics for the parallelization of the clustering step.

$N_{p}$	Time (min.)	$S(N_{p})$	*P*	1−P
**1**	508.76	1.0000	1.000	0.0000
**2**	263.26	1.9326	0.9651	0.0349
**4**	141.04	3.6071	0.9637	0.0363
**6**	101.01	5.0367	0.9618	0.0382
**8**	77.41	6.5719	0.9690	0.0310
**10**	65.22	7.8008	0.9687	0.0313

## Abbreviations

The following abbreviations are used in this manuscript:

AI	Artificial intelligence
B	Blue
BLAST	Basic Local Alignment Search Tool
CCL	Connected components labeling
CFU	Colony forming unit
DEM	Digital elevation model
DN	Digital numbers
DNA	Deoxyribonucleic acid
EXIF	Exchangeable image file format
FWHM	Full width at half maximum
G	Green
GCP	Ground control point
GEOBIA	Geographic object-based image analysis
GEOTIFF	Georreferenced Tagged Image File Format
GIS	Geographic information systems
GPIO	General purpose input output
GPS	Global positioning system
ITS	Internal transcriber spacer
LSMSS	Large scale mean shift segmentation
MDM	Minimum distance to mean
MLC	Maximum likelihood classifier
MSS	Mean shift segmentation
NCBI	National Center for Biotechnology Information
NIR	Near infrared
NDVI	Normalized difference vegetation index
NNC	Nearest neighbor classifier
OTB	Orfeo Toolbox
PA	Precision agriculture
PC	Parallelepiped classifier
PCNB	Pentachloronitrobenzene
PCR	Polymerase chain reaction
R	Red
RE	Red edge
RGB	Red, green, blue
SAM	Spectral angle mapper
UAV	Unmanned aerial vehicle
USB	Universal serial bus
VHR	Very high resolution
